# The circadian clock system’s influence in health and disease

**DOI:** 10.1186/s13073-017-0485-2

**Published:** 2017-10-31

**Authors:** Joseph T. Bass

**Affiliations:** Department of Medicine, Division of Endocrinology, Metabolism and Molecular Medicine, Northwestern University Feinberg School of Medicine, Chicago, IL USA

## Abstract

Joseph T. Bass discusses recent developments in circadian clock research and reflects on the future of the field and its potential applications to clinical medicine.

## Introduction

**Fig. 1:**
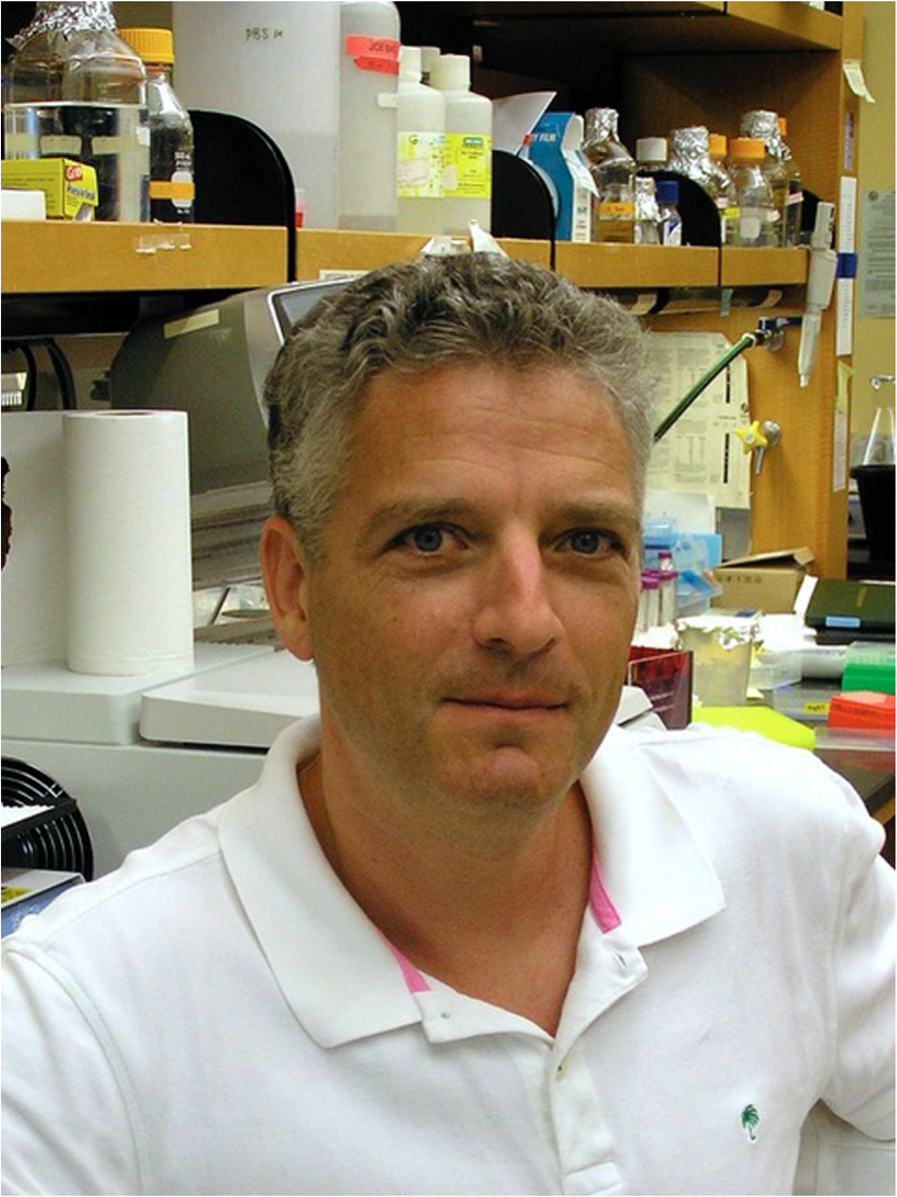
Joseph T. Bass

Joseph T. Bass (Fig. 1) is Director of the Center for Diabetes and Metabolism and Chief of Endocrinology at Northwestern University Feinberg School of Medicine. He is a leading researcher in the circadian rhythm field and the circadian clock system. In this Q&A, he shares his vision for the future of the field.

## How did you become involved in the field of circadian clock research?

My training is in medicine and the sub-specialty of endocrinology, but I also have a background in molecular biology and biochemistry. I am interested in the intersection between genes, behavior, and physiology. In the 1990s, when I was a postdoctoral fellow at the University of Chicago, there were two seminal discoveries in this field. The first was in feeding behavior with the discovery of leptin [1], and the other was in the circadian clock system, with the discovery of the mouse clock genes using forward genetics [2]. I knew from the endocrinology and clinical medicine standpoints that the circadian system was an organizing feature of physiology, especially of endocrine systems. However, the molecular underpinnings of this system and the processes involved were completely mysterious. Realizing that this discovery (the mammalian and fly circadian genes) opened up these genetic mechanisms was fascinating.

When I started up my lab at Northwestern University, my laboratory space was located next to some of the premier members of the circadian research community, including Joseph Takahashi, who had actually done the original positional cloning of the clock mutation, and Fred Turek, who worked with Joe to identify the *Clock* mutant animal, which was originally discovered in a classic genetic screen to identify mammalian clock genes. In addition to circadian defects, the first Clock mutant (called *Clock*, for “circadian rhythms of locomotor activity kaput”) exhibited endocrine abnormalities and gained more weight than regular mice. However, no systematic analysis of molecular endocrinology or biochemistry in these animals had yet been performed at the time to exploit these new genetic models. I had a hunch that temporal processes were a fundamental component of homeostatic systems, including weight and metabolic systems, and that this would be a unique entry point to elucidate the intersection between clocks and metabolism. I directed my attention towards understanding whether these animals had other endocrine abnormalities, and if so, what the underlying cause was. As each peel of the onion came off, it became clearer that this was actually a fundamental process that we were familiar with clinically, but that we had no understanding of at a molecular level. The *Clock* mutant mice, which had disorganized circadian sleep/wake behavior, also gained weight if they were challenged with a high-fat diet [3]. This finding suggested that the clock system and the related sleep/wake cycles it regulates might somehow intersect with the behavioral circuitry that controls weight homeostasis. In retrospect, this may not seem so earth shattering, but at the time most of our knowledge of weight regulation was based on the concept of nutrient homeostasis; in other words, that nutrient systems controlled weight and that abnormalities in weight or obesity were due to an abnormality in these long-term homeostatic or short-term satiety systems. I began to think that this was evidence of communication between a system that controlled a different behavior—sleep/wake behavior and photoperiodism—and these fundamental nutrient-responsive homeostatic systems.

The significantly clearer point that we stumbled upon was that although these mice had high blood sugar, they were also insulin deficient. This was really the area in which I was trained: the biology of insulin, its production, and its use as a model for how genes regulate physiology and biochemistry. The idea was that the clock genes that encoded transcription factors must be involved in regulating insulin production in the pancreatic β-cells. This led to about 10 years of work in the pursuit of proving that the clock system in the pancreas controls insulin secretion. Importantly, the tools weren’t all initially available and this was not a trivial technical feat. The link between clocks and pancreatic β-cell function was also a new observation, so the burden of proof was against us and I feel fortunate that we made the prediction accurately, and I hope it provides more opportunity to understand the fundamental regulatory control of β-cells.

## What does the 2017 Nobel Prize to Young, Rosbash, and Hall for discoveries of the molecular mechanisms controlling the circadian rhythm mean to the field and its application to medicine?

I think there are various reactions that you always have to the announcement of major prizes, but the most important one is probably a reality check. We live in a world of media saturation, but it’s not just the prizes, it’s the process of discovery that really matters. On the one hand, there are many fields in which important discoveries warrant recognition and often the choice is based on the impact on human health. On the other hand, people in this field will be pleased because it highlights the importance of this area of research; therefore, it’s welcome and gratifying. I think there are several points that provide context—the first is that Seymour Benzer and Ron Konopka showed using *Drosophila* genetics that genes control behaviors in their original experiments in the fly [4], which led to the cloning of the genes that control sleep/wake behavior and to the Nobel Prize this year. This fly work was part of a continuum of research establishing that genes control sleep/wake behavior, and that the genes involved encode transcription factors that operate through a feedback mechanism in the brain. Subsequent work showed that clock genes are widely expressed and present in nearly every cell throughout the body. These studies were the foundation for subsequent work in the mammalian system. The entry of genetic studies in the mouse really came from the discovery of the mouse clock system genes [2]. This was originally pursued by Fred Turek and Joe Takahashi, who screened and cloned these genes. All of our work is built upon the availability of mouse mutants. Conceptually, the ideas from the fly model were an integral component of the research. Regarding the genes that control behavior related to feeding, we knew that there were molecular pathways that could account for the control of body weight, and there had been a long history of molecular studies in terms of endocrine physiology. Insulin, in particular, has always been an intriguing model with which to understand molecular processes, and the idea that transcription factors underlie the regulation of β-cell function was part of my early education. This was another cornerstone of being able to bring these ideas together: recognizing that the concepts could be combined. The Nobel Prize recognizes a critical aspect of this because there has been skepticism about the validity of studies of circadian processes as some research hasn’t taken advantage of molecular and genetic approaches, and so it’s been difficult to get a definitive understanding and to forecast how this might apply to medicine, but we have this validation now. It is recognition for a field that’s not as well recognized as many other fields of medicine.

## How has the field evolved from the early model organism days and what recent advances in circadian clock research are you most excited about?

If we think of the brain, how genes modulate interactions with the environment is interesting at multiple levels. It is intriguing how the clock, in so far as it’s a genetically programmed system, responds to the environment. How the clock affects the control of behavior by the central nervous system and the processes through which peripheral tissues communicate with the brain and vice versa are also interesting. These are all prevalent themes in different experimental systems in the field. Like other fields, genome biology has been very important and, as the clock is a transcriptional mechanism, using approaches from next-generation sequencing enables a lot of unbiased hypothesis testing to understand how clocks exert these diverse control processes. Newly available technologies can be used that interrogate these mechanisms at the genome level and beyond, controlling transcription, RNA biology, protein translation, and protein processing. Each level is another area for clocks to enter. I keep thinking about the paradigm shift by which the history of physics was transformed by understanding time and its relationship to energy and mass. We don’t think of intermediary biochemical processes in terms of the dimension of time; however, if we use a train analogy then there would be time in biochemical processes as there is in a central train station controlling clocks in the brain and all the organs would have their own trains running. Thus, inside these tissues, time is something relative to the brain clock or to somebody standing inside the brain looking out. There isn’t a single discovery that I’m most excited about; it’s rather the conceptual thought experiment that we are obligated to perform. We recognize all these aspects of biological control but what if we considered how time influences this control? We should not talk about it in a vague way but should instead state that we can manipulate certain genes to answer detailed questions about how body clocks participate in physiology.

## Which diseases are linked to circadian dysfunction?

In the clinical realm of epidemiology, the association is very clear between shift work, which subjects workers to circadian disruption induced by sleep deprivation or changing sleep time, and disease [5]. Whenever we talk about clocks we should think about the implications for sleep, and that’s a complicated inter-relationship. At an epidemiological and clinical level, metabolic and endocrine systems are among the most overt in terms of the connection to a timing mechanism because glucose is metabolized differently in the day and night. Liver metabolism also varies during the day and night across many different pathways, including the detoxification pathways, the generation of sterols and other macromolecules, and the activation of mitochondria and the production of energy [6]. All these processes are alternating by day and night, and this is controlled by the circadian clock. We find that shift workers are more susceptible to diabetes and there is also a correlation with body weight and sleep time—thus, there is a rationale for focusing on the endocrine system.

There are many other examples, including asthma, which exhibits a strong association with nocturnal events, or blood pressure control, which is also strongly rhythmic as it’s controlled through the hypothalamic–pituitary–adrenal axis. The hypothalamus controls the pituitary and the adrenal gland’s production of mineralocorticoids, which are also under rhythmic control, as is the autonomic nervous system. Many of the fundamentals of physiology taught in medical school are controlled by rhythms that are 24 hour in nature, or some interval of that, but we have never used this knowledge. It’s analogous to moving from providing insulin isolated from the pig pancreas to the availability of insulin as a recombinant drug. To the end user perhaps the difference wasn’t evident, but a revolution occurred to make it possible, and there is something analogous to this currently occurring in this field. There’s an information and intellectual revolution that hasn’t yet translated into a therapeutic change because it’s pleiotropic, affects many different processes, and it’s a matter of working through where the system can be manipulated in such a way as to improve health in a certain disease. Another important area to mention is cell growth, immune signaling, and inflammation [7]. These are all processes in which there are intersections with the clock, and even the basics of what we know about aging are interlinked with clock processes [5]. Many different systems are affected; this is a conceptual entry point into a broad range of opportunities for understanding disease.

## What are the main challenges in studying the circadian clock system?

A challenge for the study of this system for the generalist and for those doing the experiments using the available tools is to be rigorous in how we think about time in different phases of the day/night cycle, as different physiologic systems are called upon to serve different regulatory processes. For instance, during sleep, the liver must produce ATP (it needs to produce substrate to maintain glucose availability for the brain) and it must integrate the storage and breakdown of energy. So, the challenge if you want to understand how the clock is affecting the system overall is to look not just at the steady state, but also to examine the system under conditions where time and the functions in the organ are varied. In endocrinology we frequently do this to try to discover whether there is a deficiency in a hormone by challenging the axis required for the hormone to elicit an effect. For example, to investigate how the adrenal gland produces cortisol we can give the recombinant form of cosyntropin and look for stimulation. Similarly, we can investigate how the liver produces glucose when it’s called on to do so. This requires us to fast an animal or to give the animal glucagon but a common mistake is to draw conclusions about liver function that don’t fill in the picture of what a timing system does. We should investigate under conditions in which the clock is directing a homeostatic process that can be tested through challenging that process. Thus, time and the directionality should be considered with respect to which arm of the homeostatic process is being investigated. Another example is the regulation of glucose levels in the blood, and there are two conditions under which we can study it. One is in response to a glucose challenge where the pancreas must produce insulin. The other is in response to insulin in which the pancreas must produce glucagon and other counter-regulatory hormones. So, to understand the alternating nutrient condition (which is either high or low glucose), we must challenge under conditions where the genes for the system are manipulated and the time of day is controlled. It’s a challenge to figure out how to test the system properly.

## What impact do you think circadian clock research will have on our understanding of diseases and does it offer potential for the early diagnosis or better prognosis of disease?

There is a lot of interest in identifying biomarkers that may change across different timeframes and which may provide a signature, for example, of how the immune system functions or how well the body can burn energy at different times of day (there are many different examples).

Another area is chronopharmacology. This describes how certain drugs are metabolized differently at different times of day and, therefore, it may be optimal when studying drug metabolism to test the levels of the drug at different times in the cycle and to take advantage of this information to adjust and manipulate drug dosing.

There are other behavioral implications, such as for optimal performance in shift work. Shift work is the extreme that we are all familiar with, but there is also a form of shift work occurring in a much more insidious way in our own lives. Computer and electronic displays emit blue light that stimulates the photo-responsive part of the eye that sets the clock, so you don’t have to work in a factory to experience the adverse effects of shift work or ‘social jetlag’. It’s a pretty prevalent rift in health and there are ways of detecting it. Clearly, one of these is to notice when you feel tired during the day or when you experience changes in your sleep/wake behavior or other patterns that you recognize as being daily routines.

Another example is that there is a greater potential for the heart to have arrhythmias at certain times of the day in people at risk of arrhythmia. All these circumstances can now be understood within the rational framework that these are rhythmic processes that evolved in a 24-hour period long before the advent of modern life.

## What aspects of the circadian clock are most likely to be implemented in medical practice? Are there promising molecular targets or is it most useful for optimizing treatment strategies, such as the timing of drug administration?

I think that the main applications are going to be in preventative medicine, diagnostics, and molecular tools to intervene in metabolic and other systems. We are not all the way there yet, but I think we will see these emerge in the next 5–10 years. We know in hospital settings that tube feedings are often at times that conflict with the endogenous circadian program and that this exacerbates insulin resistance in response to tube feedings. I often see this in the intensive care unit, where drug or nutrient infusions are provided without consideration for the clock, leading to disorganization of the circadian alignment of different systems. This is an area we should carefully consider in clinical medicine because we are potentially exacerbating problems. One of the most promising targets is cryptochrome, which is the repressor protein in the clock and a good example of a druggable molecule. Some of the proteins in the core clock itself could be targets given the correct pharmacologic opportunity. We are also interested in chemical approaches to identify, in an unbiased way, molecules that may restore the system to the setting in the event of an altered clock.

## What does the future hold for circadian system research, chronotherapy, and chronomedicine?

I think that further defining how the clock controls basic processes will continue to evolve, as well as genome biology and its downstream effectors. We really don’t know the connectivity from the clock to all the other systems that it controls or what these connections are or how they respond under perturbed conditions. In the brain, we have very sophisticated tools to question how brain centers control all sorts of functions and to elucidate exactly the interactions of the clock systems with other pathways. It is a very rich interdisciplinary field, there are many talented investigators, and there are several angles that will provide exciting new insights in both the fly and the mouse systems. It will also provide insights into sleep, which is an area where we really don’t understand much about the molecular underpinnings but which will be hugely important to reveal.
